# Acquired aortopulmonary fistula – a case report highlighting diagnostic and surgical challenges

**DOI:** 10.1093/ehjcr/ytag292

**Published:** 2026-07-14

**Authors:** Martina Vito, Valentina Scheggi, Eleonora Giovagnini, Meghi Murati, Pierluigi Stefàno

**Affiliations:** Division of General Cardiology, Careggi University Hospital, Department of Experimental and Clinical Medicine, Largo G. A. Brambilla 3, 50134 Florence, Italy; Division of Cardiovascular and Perioperative Medicine, Careggi University Hospital, Cardiothoracovascular Department, Largo G. A. Brambilla 3, 50134 Florence, Italy; Division of Internal Medicine, Careggi University Hospital, Department of Experimental and Clinical Medicine, Largo G. A. Brambilla 3, 50134 Florence, Italy; Division of General Cardiology, Careggi University Hospital, Department of Experimental and Clinical Medicine, Largo G. A. Brambilla 3, 50134 Florence, Italy; Division of Cardiac Surgery, Careggi University Hospital, Cardiothoracovascular Department, Largo G. A. Brambilla 3, 50134 Florence, Italy

**Keywords:** Case Report, Aorto-pulmonary fistula, Aortic Arch Aneurism, Pulmonary Hypertension, Right Heart Failure, Right Heart Catheterization, Multimodality Imaging, Frozen Elephant Trunk

## Abstract

**Background:**

Aorto-pulmonary fistula (APF) is a rare but potentially life-threatening condition, most often secondary to aortic surgery or dissection. This case report describes a non-iatrogenic APF caused by a chronic aortic arch aneurysm, an uncommon aetiology not widely described in the literature.

**Case Summary:**

A 68-year-old woman was admitted for severe right heart failure (RHF) secondary to pulmonary hypertension refractory to high-dose diuretics. Three months prior, aortic arch aneurysm was documented, but the cause of PH remained unclear. Targeted transthoracic echocardiography (TTE) using off-axis views revealed abnormal aorto-pulmonary flow, confirmed by dedicated CT angiography. She underwent staged open surgery, resulting in complete fistula closure and full haemodynamic recovery. Postoperative recovery was uneventful, with marked improvement in RHF, right ventricular function, and pulmonary pressures at 3 months.

**Conclusion:**

Prompt diagnosis, guided by high clinical suspicion and targeted, multimodal imaging, is essential for identifying rare and potentially life-threatening causes of pulmonary hypertension. Early diagnosis facilitates timely intervention and significantly improves patient outcomes.

Learning points

- High clinical suspicion with targeted multimodal imaging (TTE, CTA, and, when required, ETT or RHC) is key to detecting rare causes of pulmonary hypertension (e.g. APF).
- Early diagnosis is critical to enable timely surgical correction, prevent progression to advanced right heart failure, and improve perioperative and long-term outcomes.

## Introduction

Aorto-pulmonary fistula (APF) is a rare but potentially life-threatening condition, most commonly occurring as a complication of thoracic aortic aneurysm or dissection.^1,2^ It is more frequently located in the ascending aorta and in the distal portion of the aortic arch.^2^ In the majority of case reports, APF arises as a postoperative complication of cardiac surgery, most often following replacement of the aortic valve and/or ascending aorta.^3,4^ Non-iatrogenic forms are extremely rare and have been described only in isolated cases, usually associated with trauma, inflammatory aortitis, infectious aetiologies such as syphilitic aortitis^2^ or connective tissue disorders including Marfan syndrome.^5^

We report a case of non-iatrogenic APF caused by a long-standing aortic arch aneurysm, progressively eroding into the pulmonary trunk due to sustained pulsatile mechanical stress.

## Summary figure

**Table:** 

Three months before admission to our institution	First hospitalization for new-onset right heart failure with pulmonary hypertension. CT angiography showed a 6×5 cm aortic arch aneurysm and pulmonary artery ectasia; no shunt was identified. Discharged without a defined aetiology.
Current admission	Admitted with severe right heart failure, anasarca, hypotension, and tachycardia. Echocardiography revealed severe pulmonary hypertension and right ventricular dysfunction.
Diagnostic work-up	Targeted off-axis echocardiography and focused CT angiography confirmed an aortopulmonary fistula between the aortic arch and pulmonary trunk.
Surgery	Open repair with direct closure of the pulmonary trunk and aortic arch replacement (frozen elephant trunk technique).
Postoperative course	Initial haemodynamic instability requiring intensive therapy; gradual stabilization and transfer to cardiac rehabilitation.
Three-month follow-up	Clinical and haemodynamic recovery with improved right ventricular function and reduced pulmonary pressure.

## CASE REPORT

A 68-year-old woman was admitted to our institution for severe right heart failure (RHF) with multiorgan congestion requiring high-dose intravenous diuretics. Her past medical history was notable only for chronic obstructive pulmonary disease (COPD); no other significant comorbidities, cardiovascular diseases, or previous surgeries were reported. Three months earlier, she had been hospitalized for new-onset RHF associated with pulmonary hypertension (PH), presenting with progressive fatigue and exertional dyspnoea. CT-angiography (CTA) performed at the time excluded acute pulmonary embolism but revealed a 6 × 5 cm aortic arch aneurysm along its concave aspect (*Figure 1*), requiring cardiac surgery, and diffuse ectasia of the main and branch pulmonary arteries. She was discharged after optimization of medical therapy, which included guideline-directed heart failure treatment (ACE inhibitor, beta-blocker, mineralocorticoid receptor antagonist, and SGLT2 inhibitor) along with loop-diuretics. However, no definitive explanation for the PH aetiology was identified, and she was referred to a specialized outpatient clinic for further assessment.

**Figure 1:**
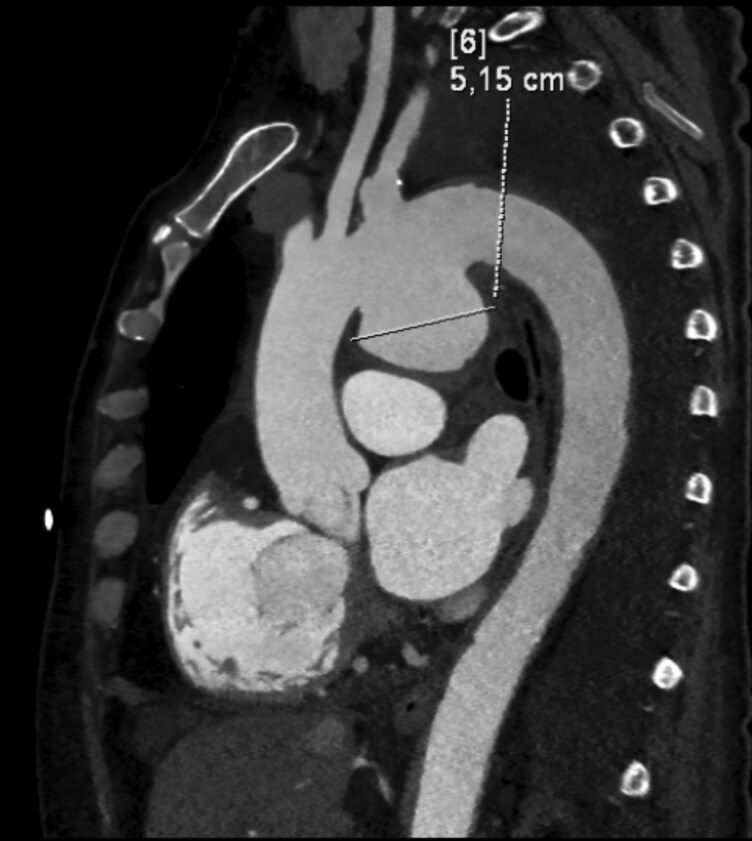
Contrast-enhanced CT angiography showing a saccular aneurysm of the aortic arch along its concavity, arising distal to the origin of the left common carotid artery, measuring 5 cm × 6 cm.

At current admission, the patient presented with severe clinical deterioration characterized by generalized anasarca and worsening dyspnoea. On physical examination, she appeared hypotensive (BP 90/40 mmHg), tachycardic (HR 115 bom), and tachypnoeic (RR 22 breaths/min) with oxygen saturation 98% on 3 L/min supplemental oxygen. Cardiovascular examination revealed a systolic murmur best heard at the mesocardium with accentuation of the second heart sound, and pulmonary auscultation revealed basal pulmonary congestion. Laboratory tests showed markedly elevated NT-proBNP levels (20 725 pg/ml; *nr* 1–125 pg/ml); impaired liver function consistent with congestive hepatopathy with AST 323 U/l, ALT 503 U/l; (*nr* 10–35 U/l), total bilirubin 4.1 mg/dl (*nr* 0.2–1.0 mg/dl), INR 1.6 (*nr* 0.8–1.2); mild anaemia with Hb 11 g/dl (*nr* 12–16 g/dl); reduction of renal function with creatinine 1.14 ml/min (*nr* 0.50–1.20 ml/min); and borderline lactate levels (2 mmol/l).

In view of her clinical and laboratory findings, heart failure therapy was maintained, and loop-diuretic therapy was intensified with high-dose intravenous continuous infusion, providing clinical benefit; subsequent attempts to taper were unsuccessful, reflecting advanced diuretic-dependent RHF.

Transthoracic echocardiography (TTE) demonstrated worsening PH (PASP 70 mmHg), severe right ventricular (RV) dilatation (RVD1 50 mm) and dysfunction (TAPSE 14 mm), and severe tricuspid regurgitation, with no significant structural or Doppler abnormalities of the left heart. Based on prior radiologic imaging, an aortopulmonary shunt was suspected as the underlying cause, despite the previous CT being negative.

Targeted TTE, using off-axis PLAX views, demonstrated abnormal flow from the aorta to the pulmonary artery, consistent with a shunt. A dedicated CTA of the thoracic aorta subsequently confirmed a fistulous communication between the aneurysmal aortic arch and the pulmonary trunk.

The patient underwent open surgical repair consisting of direct closure of the fistulous tract by suturing the pulmonary trunk, combined with aortic arch replacement using the frozen elephant trunk technique with a hybrid graft (Artivion E-Vita Open NEO 28–180). Postoperatively, she experienced transient haemodynamic instability requiring intensive support but gradually stabilized and was transferred to structured cardiac rehabilitation. At discharge, heart-failure therapy was continued, with gradual tapering of diuretics according to clinical status.

At three-month follow-up, she showed marked clinical improvement, RV functional recovery, mild-to-moderate tricuspid regurgitation, and a significant reduction in pulmonary pressures. Heart failure therapy was safely discontinued, with only antihypertensive treatment (ramipril and bisoprolol) maintained, highlighting that early diagnosis and correction of the underlying cause are crucial to PH management, according to ESC/ERS guidelines.

## Discussion

Pathophysiologically, APF creates a left-to-right shunt, leading to PH and worsening RHF.^6^ In most reports, clinical presentation is abrupt and dramatic, often with chest pain, rapid deterioration of RV function, and, in severe cases, haemodynamic collapse. By contrast, subacute presentation is uncommon and may manifest with subtle, non-specific symptoms such as profound fatigue or exertional dyspnoea, which may be misattributed to more common conditions, particularly in the absence of predisposing factors for APF.^7^ Consequently, diagnostic delay is possible and may worsen perioperative outcomes.

Early recognition of APF is challenging due to its rarity—seldom considered in the differential diagnosis of RHF with PH—and limitations of first-line imaging. Standard TTE may easily miss APF unless a focused assessment is performed under a high index of suspicion, often requiring off-axis views and experienced operators.^7^

This case illustrates these diagnostic challenges. The first hospitalization resulted in patient’s discharge with the diagnosis of RHF secondary to PH, yet the underlying aetiology remained unclear despite documentation of a large aortic arch aneurysm. As a result, a definitive diagnosis was established three months later, when the patient was readmitted to our institution with severe clinical deterioration due to worsening RHF, which is a typical consequence of delayed diagnosis and treatment of PH, which may lead to refractory heart failure and, in severe cases, cardiogenic shock and multiorgan failure. Moreover, severe RHF markedly increases surgical risk, representing a critical concern when PH can only be definitively treated surgically, as in this case.

This highlights the need for a comprehensive diagnostic approach to identify the aetiology of PH, combining careful clinical assessment (including cardiac auscultation to detect the presence of a continuous systolic–diastolic murmur, best heard over the left upper sternal border), evaluation of predisposing conditions for uncommon aetiologies, including thoracic aortic pathology for APF, and meticulous interpretation of non-invasive imaging (TTE, CTA). If inconclusive, right heart catheterization (RHC) should be considered early in the diagnostic work-up as it provides a detailed haemodynamic assessment of PH, potentially revealing the presence of step-up in oxygen saturation indicative of a left-to-right shunt that enables timely and appropriate intervention.^8^ In this case, RHC at initial admission might have allowed earlier diagnosis (preventing further RHF worsening) and cardiac surgery in more favourable conditions.

At the admission to our centre, this diagnostic approach proved decisive. Re-evaluation of the prior CTA raised suspicion of APF, prompting focused echocardiography. Off-axis PLAX view demonstrated turbulent flow within the pulmonary trunk and abnormal communication with the aorta (*Figure 2*), strongly suggestive of a shunt.

**Figure 2:**
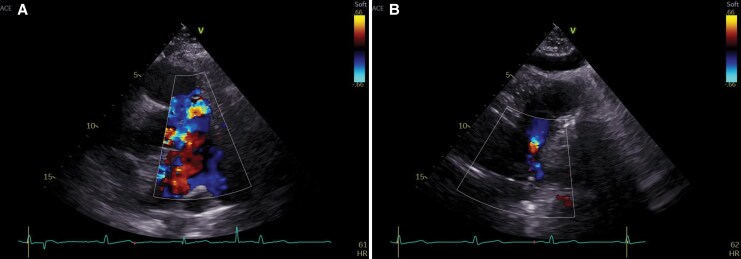
Off-axis parasternal short-axis echocardiographic views on Colour-Doppler demonstrating turbolent flow within the pulmonary trunk in Panel A and an abnormal communication between the aortic arch and the pulmonary trunk in Panel B, consistent with a left-to-right shunt.

Subsequent thoracic aortic CTA confirmed the diagnosis; where explicitly stating APF suspicion in the referral enabled focused evaluation (*Figure 3, 4*). Although CTA represents the diagnostic gold standard for APF, small fistulas may be easily missed unless specifically investigated, as initially occurred in this case, resulting in diagnostic delay.

**Figure 3:**
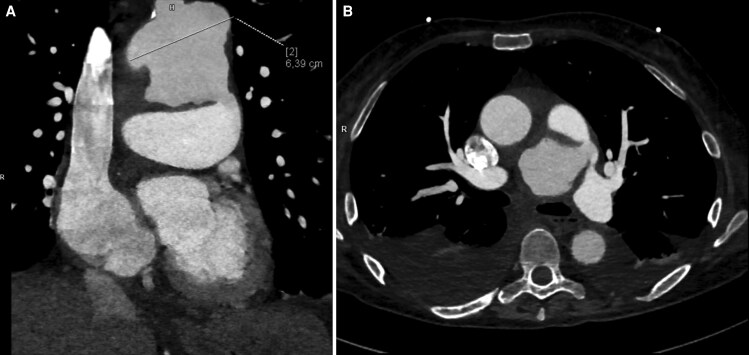
Two-dimensional CT angiography of the aorta demonstrates a small fistulous tract between the aortic arch aneurysm and the pulmonary trunk. Coronal Plane in Panel A and Axial Plane in Panel B.

**Figure 4:**
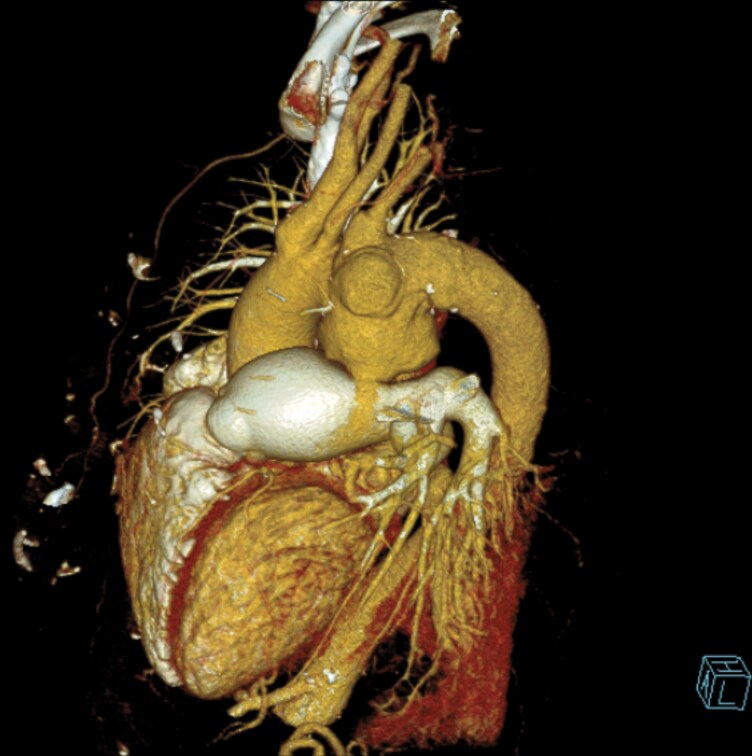
Three-dimensional computed tomography reconstruction demonstrating the aortopulmonary fistula between the aortic arch and the pulmonary trunk.

Unlike many published cases requiring multimodal imaging—including transoesophageal echocardiography (*Figure 5*) and RHC^9^— in this case APF was promptly identified through focused non-invasive evaluation, avoiding unnecessary invasive procedures.

**Figure 5:**
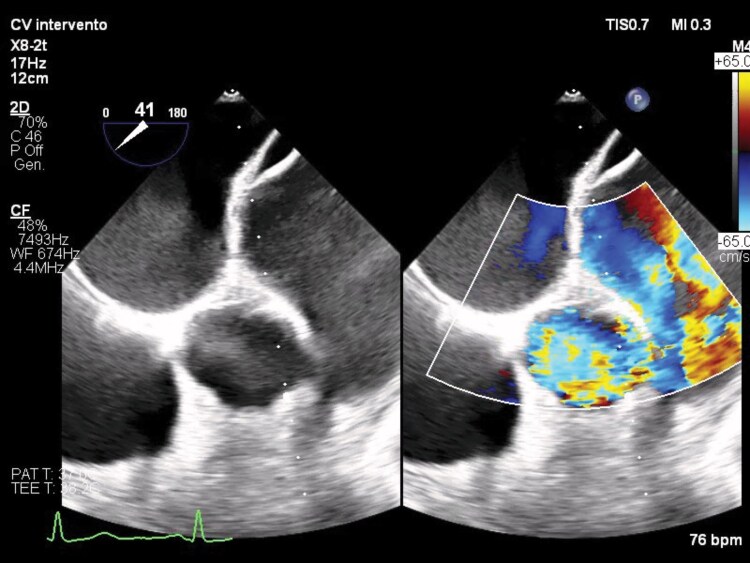
Intraoperative transoesophageal echocardiography in a mid-oesophageal short-axis view above the aortic valve plane. It shows abnormal flow between the aorta and the pulmonary trunk.

Open surgical repair remains the gold-standard treatment,^8^ allowing definitive closure of the fistulous tract, either by primary suture or prosthetic patch,^10^ with concomitant aortic repair. Surgery provides durable results with low recurrence but is invasive, with higher periprocedural risk and longer recovery. Early mortality is influenced by age, comorbidities, aortic arch involvement, dissection, and severe ventricular dysfunction.^8^ In high-risk patients, percutaneous techniques —including coil embolization and endovascular stent-grafts—reduce procedural risk and recovery time but may not achieve complete closure and are unsuitable for complex or multiple defects.^10,11^

In this case, conventional surgery was required due to anatomical complexity. Definitive repair included direct suture closure of the pulmonary trunk reinforced with pledgets, combined with aortic arch replacement using the frozen elephant trunk technique and a hybrid graft (Artivion E-Vita Open NEO 28–180).

## Conclusion

This case highlights an unusual subacute APF presentation, contrasting with the acute and catastrophic courses usually reported in the literature. Prompt recognition was achieved through targeted imaging, guided by high clinical suspicion in a patient with unexplained severe RHF and PH refractory to maximal therapy, avoiding unnecessary invasive procedures. Early diagnosis is crucial, as sustained RV overload substantially impairs cardiovascular outcomes and adversely affects perioperative risk. Definitive open surgery provided complete fistula closure, normalization of pulmonary pressures, and recovery of RV function, underscoring that identification and correction of the underlying cause remains central to PH management, in line with ESC/ERS guidelines.

## Data Availability

The data underlying this article will be shared on reasonable request to the corresponding author.

## Lead author biography

Valentina Scheggi, MD, is an internist at Careggi University Hospital, Florence, Italy. Martina Vito is a Cardiology Resident at Careggi University Hospital, Florence, Italy. Both authors are involved in clinical care and cardiovascular research.

## Notes

**Consent:** The authors confirm that witnessed verbal consent for submission and publication of this case report, including images and associated text, has been obtained from the patient. This has been discussed with the editors.
